# Radiation reaction as an energy enhancement mechanism for laser-irradiated electrons in a strong plasma magnetic field

**DOI:** 10.1038/s41598-019-53644-x

**Published:** 2019-11-20

**Authors:** Z. Gong, F. Mackenroth, X. Q. Yan, A. V. Arefiev

**Affiliations:** 1 SKLNPT, KLHEDP and CAPT, School of Physics, Peking University, Beijing, 100871 China; 2 Center for High Energy Density Science, The University of Texas at Austin, Austin, TX 78712 USA; 3 Max Planck Institute for the Physics of Complex Systems, 01187 Dresden, Germany; 4 Department of Mechanical and Aerospace Engineering, University of California at San Diego, La Jolla, CA 92093 USA; 5 Center for Energy Research, University of California at San Diego, La Jolla, CA 92093 USA

**Keywords:** Laser-produced plasmas, Plasma-based accelerators

## Abstract

Conventionally, friction is understood as a mechanism depleting a physical system of energy and as an unavoidable feature of any realistic device involving moving parts. In this work, we demonstrate that this intuitive picture loses validity in nonlinear quantum electrodynamics, exemplified in a scenario where spatially random friction counter-intuitively results in a highly directional energy flow. This peculiar behavior is caused by radiation friction, i.e., the energy loss of an accelerated charge due to the emission of radiation. We demonstrate analytically and numerically how radiation friction can dramatically enhance the energy gain by electrons from a laser pulse in a strong magnetic field that naturally arises in dense laser-irradiated plasma. We find the directional energy boost to be due to the transverse electron momentum being reduced through friction whence the driving laser can accelerate the electron more efficiently. In the considered example, the energy of the laser-accelerated electrons is enhanced by orders of magnitude, which then leads to highly directional emission of gamma-rays induced by the plasma magnetic field.

## Introduction

For an accelerated particle of charge *q* and mass *m* the main energy dissipation, or friction, is the continuous emission of electromagnetic radiation, referred to as *radiation friction* (RF). The energy loss per unit time is given by^1^

1 $$P=\frac{2{q}^{2}{\varepsilon }^{2}}{3{m}^{4}{c}^{7}}{(\frac{d{p}^{\mu }}{dt})}^{2},$$

where *c* is the speed of light, *p*^*μ*^ is the particle relativistic momentum, *ε* is its energy, and *t* is time. Several structural peculiarities of RF were described, e.g., mathematically ill-behaved particle dynamics^2^ or the need for charge renormalization in classical electrodynamics^3^. For RF to reduce a particle’s energy *ε* at a rate corresponding to the particles’ momentum change due to acceleration, the emitted power $P=d\varepsilon /dt=(d\varepsilon /d|{\boldsymbol{p}}|)(d|{\boldsymbol{p}}|/dt)$, with the particle momentum ***p***, needs to match the accelerating force, resulting in $|{{\boldsymbol{F}}}_{RF}|=|dp/dt|\sim 3{m}^{4}{c}^{8}/2{\varepsilon }^{2}{q}^{2}$. Early studies deemed this regime of *instantaneous RF* unreachable in a lab, as it would require accelerating electromagnetic fields ${E}_{RF}\ge {F}_{RF}/|e|\sim {10}^{15}$ V/m even for an electron (the lightest particle) at mildly relativistic energies of ${\varepsilon }_{e}\sim 100\,{m}_{e}{c}^{2}$. Such field strengths, however, are becoming increasingly available due to the advent of ultra-high intensity lasers^4,5^ providing intensities ${I}_{L}\gtrsim {10}^{22}\,\,{\rm{W}}/{{\rm{cm}}}^{2}$ at optical wavelengths (*λ*_*L*_ ~ 1 *μ*m), corresponding to electric fields ${E}_{L}\gtrsim {10}^{15}$ V/m^6^, with facilities aiming at higher fields under construction^7,8^, sparking renewed interest in instantaneous RF^9–11^. At these facilities, even experimental observation of the instantaneous RF became possible^12,13^ in the random energy loss of a laser-accelerated relativistic electron bunch when scattered by a high-power laser pulse. Similar setups were investigated in a series of theoretical studies^14–26^, indicating the acceleration of electrons as an important field of application for high-intensity laser facilities^27–30^.

The radiation friction has been actively studied due to its non-trivial and sometimes unexpected impact on electron dynamics in strong laser fields^31–36^. One unexpected effect revealed through an exact solution of the Landau-Lifshitz equation^37^ is the energy increase of an electron irradiated by a plane electromagnetic wave^38–40^. Even though this is a profound result, its impact on laboratory studies of high-intensity laser plasma interactions is limited by the requirement of a large distance and a large transverse spread needed to accelerate electrons to high energies.

**Figure 1:**
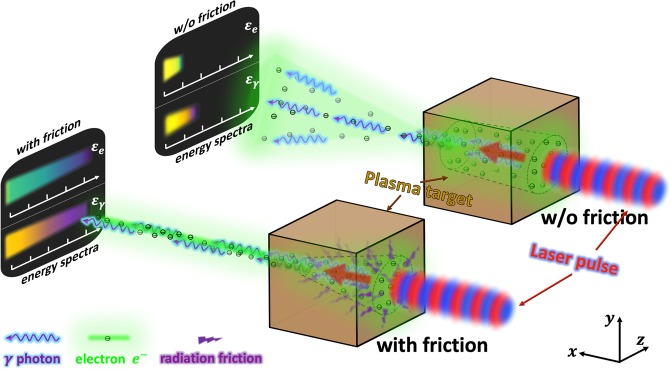
Schematic diagram summarizing the effect of the radiation friction in laser-irradiated plasmas. The green dots are the laser-irradiated electrons, the blue wavy lines are the emitted photons, and the purple fragments are the radiation friction force. Red arrows indicate the direction of the electron beam’s acceleration and the black dashed lines mark the location of the magnetic boundary associated with the plasma magnetic field that limits transverse electrons oscillations. Spectra of electron and photon energies, *ε*_*e*_ and *ε*_*γ*_, (black panels to the left) and their beam divergence (cones left of plasma targets) visualize the pronounced enhancement in peak energies and beam collimation due to the radiation friction.

In this work, we examine the dynamics of laser-irradiated electrons in the presence of a strong quasi-static magnetic field that naturally arises in a dense plasma irradiated by an ultra-high intensity laser^41–43^. We demonstrate analytically and numerically how the radiation friction dramatically enhances the electron energy gain in this setup by reducing initial transverse electron momentum and enabling a subsequent efficient energy transfer from the laser field to the electron, as schematically shown in Fig. 1. In contrast to the already discussed energy enhancement in a vacuum, the electrons are accelerated without an increase of their transverse displacement, making it easier to realize the described regime experimentally. A direct benefit of the transverse electron confinement is that the accelerated electrons are directed forward with a small angular spread, which then leads to highly directional gamma-ray emission induced by the plasma magnetic field.

## Main Model

This work is motivated by a regime of light-matter interaction where an ultra-high intensity laser pulse is able to propagate through a dense plasma and drive a quasi-static magnetic filament^41^. The laser pulse hitting a solid target quickly ionizes the material, turning it into a plasma with an electron density *n*_*e*_. We consider densities close to the critical threshold ${n}_{c}=\pi {m}_{e}{c}^{2}/({\lambda }_{L}^{2}{e}^{2})$, above which a low-intensity laser pulse with a vacuum wavelength *λ*_*L*_ cannot propagate into the plasma. In an ultra-intense laser pulse, electrons oscillate relativistically, yielding an effective mass increase that extends the cutoff density to ${n}_{c}{\bar{\varepsilon }}_{e}/{m}_{e}{c}^{2}\approx {a}_{0}{n}_{c}$. Here *a*_0_ is the normalized laser amplitude defined in terms of the laser intensity and wavelength as ${a}_{0}\equiv \sqrt{{I}_{L}[{\rm{W}}/{{\rm{cm}}}^{2}]/1.37\times {10}^{18}}{\lambda }_{L}[\mu m].$ The described effect is referred to as the relativistically induced transparency^44–48^. It makes it possible for an intense laser pulse to propagate through a dense plasma. The relativistic transparency is important at ${I}_{L}\gg {10}^{18}\,{\rm{W}}/{{\rm{cm}}}^{2}$ for *λ*_*L*_ ~ 1 *μ*m or, equivalently, at ${a}_{0}\gg 1$.

Particle-in-cell simulations performed by multiple groups have shown that this regime enables generation of strong quasi-static magnetic fields^49–53^. As the laser pulse propagates through the plasma, it pushes the electrons forward and drives a longitudinal current. The current density scales as *n*_*e*_, so it can be greatly enhanced by leveraging the relativistic transparency to propagate the laser in a plasma with *n*_*e*_ ≥ *n*_*c*_. The longitudinal current is typically distributed over the cross-section of the laser beam, whereas the return current is concentrated at the periphery of the beam. The feature that is of central importance to the present manuscript is the magnetic field that is generated and sustained by the laser-driven current. The field is azimuthal and it forms a magnetic filament co-located with the laser beam.

Additional studies have shown that the laser propagation becomes unstable in a dense relativistically transparent target^54^, which makes the direction of the laser beam propagation unpredictable. One way to suppress the instability while retaining the advantages of laser propagation through a dense plasma is to use structured targets that provide optical guiding to the laser pulse. Here we consider the design where the target consists of a cylindrical channel that is filled with a material that becomes more transparent than the bulk when irradiated by a high intensity laser beam. The channel serves as an optical wave-guide to the beam that is focused at the channel entrance. Structured targets with an initially empty channel have already been used experimentally to achieve greater control over laser interactions with solid-density targets^55^. Advanced target manufacturing facilities are also able to produce solid targets of variable density using the *in situ* polymerization technique^56^. The pore and thread structures are sub-micron, so a relatively homogeneous plasma with *n*_*e*_ ≥ 0.9*n*_*c*_ has been achieved in experiments with high-intensity lasers^57^. It is challenging but feasible to manufacture targets with pre-filled channels, so our study is performed in anticipation of this capability becoming available for experiments at high intensity laser facilities.

The effect of the radiation friction considered here is directly linked to the electron dynamics in the combined laser and plasma fields present in the channel of a structured target^41,43^. We make use of a simplified model capturing all essential physics, as demonstrated in detailed kinetic plasma simulations^41–43^. These simulations motivate the following fundamental assumptions underlying the model: (i) Since transverse electron oscillations are confined narrower than the width of the laser beam and the laser’s phase fronts inside the channel are near-flat, the electron effectively experiences it as a plane wave. (ii) Since laser beam diffraction is suppressed in structured targets^41,43^, the laser maintains its peak amplitude over distances much longer than its Rayleigh range. (iii) Dephasing between the electrons and the laser is primarily determined by the longitudinal electron velocity *v*_*x*_ whence we neglect deviations of the phase velocity *v*_*ph*_ from *c* that arise from the laser propagation through a finite-width channel filled with a plasma. It has been shown that the relativistically induced transparency significantly reduces the effect of the plasma on *v*_*ph*_^30^. (iv) The laser drives a strong, uniform longitudinal electron current density *j*_0_ < 0, with the return current flowing on the periphery of the channel. (v) The quasi-static radial electric field in the channel is much weaker than the azimuthal magnetic field due to the transverse ion mobility that reduces the charge separation caused by the laser beam^42^. (vi) The electrons are injected into the laser beam by the transverse oscillating laser electric field, so they initially have a large transverse momentum comparable to *a*_0_*m*_*e*_*c*^41,43^. (vii) Binary collisions and feedback of the accelerated electrons on the bulk plasma dynamics are negligible. Consequently, the electron dynamics can be modeled by considering a single electron in a combined plane electromagnetic wave representing the laser and a static azimuthal magnetic field representing the plasma magnetic field.

In summary, the key input parameters in our test-electron model formulated in this section are the normalized laser amplitude *a*_0_, channel current density *j*_0_, and the initial on-axis electron momentum *p*_0_.

## Energy Gain Estimate

We model the laser pulse as a linearly polarized electromagnetic wave propagating along the *x*-axis. Its electric and magnetic fields are given by

2 $${{\boldsymbol{E}}}_{wave}=-\,\frac{{m}_{e}c}{|e|}\frac{\partial {{\boldsymbol{a}}}_{wave}(\xi )}{\partial t},$$

3 $${{\boldsymbol{B}}}_{wave}=\frac{{m}_{e}{c}^{2}}{|e|}\nabla \times {{\boldsymbol{a}}}_{wave}(\xi ),$$

where $\xi \equiv (ct-x)/{\lambda }_{L}$ is the phase variable and ${{\boldsymbol{a}}}_{wave}(\xi )={{\boldsymbol{e}}}_{y}{a}_{0}\,\sin \,(2\pi \xi )$ is the normalized vector potential, with ***e***_*y*_ being a unit vector. The laser electric field in this setup is polarized along the *y*-axis. The quasi-static magnetic plasma field sustained by the constant current density *j*_0_ can be written as

4 $${{\boldsymbol{B}}}_{channel}=\frac{{m}_{e}{c}^{2}}{|e|}\nabla \times {{\boldsymbol{a}}}_{channel},$$

where ${{\boldsymbol{a}}}_{channel}={{\boldsymbol{e}}}_{x}\alpha ({y}^{2}+{z}^{2})/{\lambda }_{L}^{2}$. Here we introduced a dimensionless current density

5 $$\alpha \equiv -\,\pi {\lambda }_{L}^{2}{j}_{0}/{J}_{A}$$

that is expressed in terms of the Alfvén current ${J}_{A}\equiv {m}_{e}{c}^{3}/|e|$.

The laser pulse tends to drive flat electron trajectories in the laser polarization plane^43^, so from now on we set *z* ≡ 0 and study electron motion in the (*x*, *y*)-plane only. In the combined fields, ${\boldsymbol{E}}={{\boldsymbol{E}}}_{wave}$ and ${\boldsymbol{B}}={{\boldsymbol{B}}}_{wave}+{{\boldsymbol{B}}}_{channel}$, the electron dynamics with radiation friction (RF) taken into account are governed by the equations

6 $$\frac{d{\boldsymbol{p}}}{dt}=-\,|e|{\boldsymbol{E}}-\frac{c|e|}{{\varepsilon }_{e}}[{\boldsymbol{p}}\times {\boldsymbol{B}}]-{F}_{RF}\frac{{\boldsymbol{p}}}{|{\boldsymbol{p}}|},$$

7 $$\frac{d{\boldsymbol{r}}}{dt}=\frac{{c}^{2}{\boldsymbol{p}}}{{\varepsilon }_{e}},$$

where ***r*** and ***p*** are the electron position and momentum, *t* is the time and

8 $${F}_{RF}=\frac{\kappa {\varepsilon }_{e}^{2}{ {\mathcal E} }^{2}}{{m}_{e}^{2}{c}^{4}}$$

quantifies the impact of RF where

9 $$\kappa =8{\pi }^{2}{e}^{2}/3{\lambda }_{L}{m}_{e}{c}^{2}\approx 7.4\times {10}^{-8}.$$

We consider an electron that starts its motion on the axis of the channel at time *t*_0_ with a large transverse momentum, i.e., *y*(*t*_0_) = 0, ${\boldsymbol{p}}({t}_{0})=(0,{p}_{y,0}\gg {m}_{e}c,0)$. Then, since we study relativistic motion we retain only terms of leading order in ${\varepsilon }_{e}\gg {m}_{e}{c}^{2}$ in the dynamical RF parameter which can then be expressed as^3^

10 $${ {\mathcal E} }^{2}={(\frac{e}{{m}_{e}c{\omega }_{L}})}^{2}{({{\boldsymbol{E}}}_{\perp }+\frac{c}{{\varepsilon }_{e}}[{\boldsymbol{p}}\times {\boldsymbol{B}}])}^{2},$$

where ${{\boldsymbol{E}}}_{\perp }={\boldsymbol{E}}-{\boldsymbol{p}}({\boldsymbol{pE}})/{{\boldsymbol{p}}}^{2}$ is the electric field component perpendicular to the electron momentum. Combining the *x* and *y* components of Eq. (6), approximating the momentum as ${\boldsymbol{p}}\approx {\varepsilon }_{e}/c$, and taking into account that ${{\boldsymbol{a}}}_{wave}$ is a function of *ξ* alone, we find that

11 $$\frac{d(R+{a}_{channel})}{dt}=-\,{F}_{RF}\frac{d\xi }{dt},$$

where we introduced the *dephasing rate* $R=(d\xi /dt){\varepsilon }_{e}{\lambda }_{L}/{m}_{e}{c}^{3}=({\varepsilon }_{e}-c{p}_{x})/{m}_{e}{c}^{2}$ quantifying the change of *ξ* at the electron’s instantaneous position. Hence, for vanishing RF (*F*_*RF*_ → 0) the quantity $R+{a}_{channel}={\rm{const}}{\rm{.}}+{\mathscr{O}}({F}_{RF})$ is an integral of motion, which, in turn, implies that the motion of a relativistic electron with the above introduced initial conditions is confined to transverse displacements smaller than the *magnetic boundary*

12 $$y\le {y}_{MB}\,:\,={\lambda }_{L}\sqrt{\frac{{p}_{y,0}}{{m}_{e}c\alpha }}.$$

Taking into account RF, however, one finds that for the same initial conditions the magnetic boundary shrinks as a function of time according to

13 $${y}_{MB}(t)={\lambda }_{L}\sqrt{\frac{{p}_{y,0}}{{m}_{e}c\alpha }-{\int }_{\xi ({t}_{0})}^{\xi (t)}d\xi \,\frac{{F}_{RF}}{\alpha }}.$$

The maximum channel potential ${{\boldsymbol{a}}}_{channel}$ that can be sampled by the electron also decreases with time and it is limited by $|{{\boldsymbol{a}}}_{channel}|\le |{{\boldsymbol{a}}}_{channel}[{y}_{MB}(t)]|$.

We now turn to studying the electron energy gain. From Eq. (6) we see that it is given by the balance between acceleration and deceleration in the laser field and losses to radiation friction

14 $$\frac{d{\varepsilon }_{e}}{dt}=\sum _{i=x,y,z}\,\frac{{p}_{i}{c}^{2}}{{\varepsilon }_{e}}\frac{d{p}_{i}}{dt}=\frac{{m}_{e}{c}^{3}{\omega }_{L}}{{\varepsilon }_{e}}({p}_{y}\,\cos \,(2\pi \xi ){a}_{0}-|{\boldsymbol{p}}|{F}_{RF}).$$

The first term on the right-hand side is the energy exchange between the electron and the transverse laser electric field. It depends on the relative orientation of the transverse electron velocity and the electric field. If the radiation friction is completely negligible, then the rate of the energy gain in an optical laser field (*λ*_*L*_ = 1 *μ*m) is limited by

15 $$\frac{d{\varepsilon }_{e}}{dt}\lesssim {\omega }_{L}{a}_{0}{m}_{e}{c}^{2}\sim {10}^{-3}\,{a}_{0}[\frac{{\rm{GeV}}}{{\rm{fs}}}].$$

In the absence of the magnetic field, the electron is able to maintain the rate given by Eq. (15) only over a very short time interval due to its rapid dephasing, $R\gg 1$. As the electron slips with respect to the laser wave-fronts, the energy gain changes to an energy loss. The resulting maximum energy remains comparable to the initial electron energy. The azimuthal magnetic field can fundamentally alter the energy exchange with the wave by deflecting the electron radially and thus allowing the transverse electron velocity to remain anti-parallel to the transverse laser electric field. In this regime, the electron continues to gain energy as it slips with respect to the laser pulse. This regime leads to a significant energy gain, but it can only be achieved for a relatively small *p*_*y*,0_^43^.

The magnetic field enhances the energy gain due to the deflections only if the electron travel time between the magnetic boundaries at *y* = *y*_*MB*_ and at *y* = −*y*_*MB*_ is comparable to the period of electric field oscillations at the electron location. The travel time is proportional to *y*_*MB*_ that in turn scales as $\sqrt{{p}_{y,0}}$. The consequence of this scaling is that electrons that are injected into the channel with a large transverse momentum are unable to undergo the energy enhancement^43^. However, the radiation friction causes the magnetic boundary to contract and, as a result, electrons can enter the energy enhancement regime regardless of their initial transverse momentum. We will numerically demonstrate this in the next section of the paper. Our goal here is to estimate how much energy the electrons can gain once the conditions for that become favorable.

To estimate the maximum energy the electron can gain we need an explicit expression for ${\mathcal E}$, which requires detailed knowledge of the electron motion in the (*x*, *y*)-plane. To this end, we note that once the electron is accelerated in the laser’s propagation direction its velocity will only make a small angle $\theta \ll 1$ with the *x*-axis. Taking into account that the electron is ultra-relativistic, we can write ${p}_{y}=p\,\sin \,(\theta )\approx {\varepsilon }_{e}\theta /c$. We use this expression in Eq. (14) to find that the energy gain is limited by $d{\varepsilon }_{e}/dt\lesssim {a}_{0}{m}_{e}{c}^{2}\omega \theta -\kappa {\varepsilon }_{e}^{2}{ {\mathcal E} }^{2}/{m}_{e}{c}^{2}$. We now distinguish the following two cases: (i) If the electron radiates energy mainly due to the laser field’s action, then after some algebra one finds from Eq. (10) that

16 $${ {\mathcal E} }^{2}\approx { {\mathcal E} }_{laser}^{2}\,:\,=({\theta }^{4}+{\gamma }^{-4}){a}_{0}^{2}/4.$$

(ii) If, on the other hand, the radiative losses are dominated by the channel magnetic field one finds from Eq. (10) that

17 $${ {\mathcal E} }^{2}\approx { {\mathcal E} }_{channel}^{2}\,:\,={(e|{{\boldsymbol{B}}}_{channel}|/{m}_{e}c\omega )}^{2}\le {a}_{MB}^{2}(t),$$

where

18 $${a}_{MB}=|e{{\boldsymbol{B}}}_{channel}({y}_{MB})|/{m}_{e}c\omega $$

is the strength of normalized plasma magnetic field at the magnetic boundary *y*_*MB*_(*t*).

The role of the plasma magnetic field depends on *θ*, since the contribution of the laser electric and magnetic fields drops as the electron motion becomes more forward directed. This motivates us to introduce a critical angle, defined as

19 $${\theta }_{B}\,:\,=\sqrt{2{a}_{MB}/{a}_{0}}.$$

It follows from Eqs. (16) and (17) that ${ {\mathcal E} }^{2}$ and thus the radiation friction are determined by the channel magnetic field for *θ* < *θ*_*B*_. At large angles, *θ* ≥ *θ*_*B*_, the radiative losses are determined by the laser field. The energy gain is then given by ${d{\varepsilon }_{e}/dt|}_{laser}\approx 2\pi {a}_{0}{m}_{e}{c}^{2}\theta [1-\kappa {\varepsilon }_{e}^{2}{\theta }^{3}{a}_{0}/8\pi {m}_{e}^{2}{c}^{4}]$. Thus, the electron can gain energy only for

20 $$\theta \le {\theta }_{laser}\,:\,={(\frac{8\pi {m}_{e}^{2}{c}^{4}}{\kappa {a}_{0}{\varepsilon }_{e}^{2}})}^{\frac{1}{3}}.$$

On the other hand, the radiative losses are determined by the channel magnetic field at *θ* ≤ *θ*_*B*_, with the energy gain given by ${d{\varepsilon }_{e}/dt|}_{laser}\approx {m}_{e}{c}^{2}(2\pi {a}_{0}\theta -\kappa {\gamma }^{2}{a}_{MB}^{2})$. Consequently, the electron can gain energy in this regime only as long as

21 $$\theta \ge {\theta }_{channel}\,:\,=\frac{\kappa {a}_{MB}^{2}{\varepsilon }_{e}^{2}}{2\pi {m}_{e}^{2}{c}^{4}{a}_{0}}.$$

The qualitative difference between Eqs. (20) and (21) is that at *θ* > *θ*_*B*_ the electron can gain energy only when reducing its propagation angle, whereas at *θ* < *θ*_*B*_ the electron can gain energy only when increasing its propagation angle. This difference indicates that independently of its initial value the electron propagation angle always tends to *θ*_*B*_. Such an asymptotic behavior, in turn, implies the existence of an upper boundary for the electron energy beyond which further energy gain is prohibited due to radiative losses:

22 $${\varepsilon }_{e}^{{\rm{\max }}}={m}_{e}{c}^{2}{(\frac{8{\pi }^{2}{a}_{0}}{{\kappa }^{2}{a}_{MB}^{3}})}^{\frac{1}{4}}.$$

This expression for ${\varepsilon }_{e}^{{\rm{\max }}}$ is obtained by setting *θ*_*laser*_ = *θ*_*channel*_ in Eqs. (20) and (21) and solving for the electron energy.

**Figure 2:**
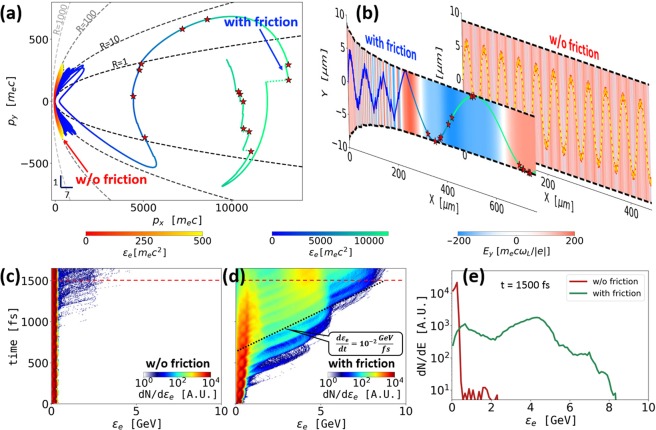
Energy enhancement by the radiation friction. (**a**) Electron trajectories in the momentum-space with (blue-green) and without (red-yellow) the radiation friction. The stars mark photon emissions with *ε*_*γ*_ > 50 MeV. (**b**) Electron trajectories in the (*x*, *y*) plane with the energy color-coding the same as in (**a**). The dashed curves show the magnetic boundary. The background color is the amplitude of the transverse laser electric field at the electron location. (**c**,**d**) Time-resolved electron spectra with and without the radiation friction. (**e**) Electron energy spectra at *t* = 1500 fs [dashed red lines in (**c**,**d**)].

In order to apply Eq. (22) to estimate the maximum electron energy gain in simulations, one should treat *a*_*MB*_ as a constant. The value must be set to that at the start of the energy enhancement process, which is likely to be lower than *a*_*MB*_ at the beginning of the simulations when the energy gain is not possible. It is shown in Sec. IV that the contraction of the magnetic boundary significantly slows down at the onset of the energy enhancement (see Fig. 2b).

Equation (22) provides an upper estimate for the electron energy in the case when the radiation friction force is included into the equations of motion. In our analysis, the primary channel for the energy losses is the radiation friction. It is important to stress that the electron energy gain can also be limited without the energy losses associated with the radiation friction. A detailed analysis similar to that presented in ref.^58^ is required to determine the corresponding maximum electron energy.

## Results

The estimate derived in the previous section states that, when the radiation friction is taken into account, an electron inside the plasma channel can gain energy up to

23 $${\varepsilon }_{e}^{{\rm{\max }}}\approx 5.6{(\frac{{a}_{0}}{{a}_{MB}^{3}})}^{\frac{1}{4}}\,{\rm{GeV}},$$

where *a*_*MB*_ is the maximum strength of the normalized channel magnetic field experienced by the electron and *κ* ≈ 7.4 × 10^−8^ is a constant calculated for *λ*_*L*_ = 1 *μ*m. The energy gain is predicted to occur after the magnetic boundary contracts sufficiently due to the radiation friction to make the travel time between the magnetic boundaries comparable to period of laser oscillations at the electron location.

In order to investigate this regime, we first consider a single electron with *p*_*y*,0_ = 100 *m*_*e*_*c* irradiated by a laser pulse with a normalized amplitude *a*_0_ = 200 and a wavelength *λ*_*L*_ = 1 *μ*m. The laser parameters correspond to an intensity of *I*_*L*_ ≈ 5 × 10^22^ W/cm^2^ that is well within the reach of next-generation laser facilities^7^. We set the normalized current density to *α* = 1.2, which corresponds to a 3.2 MA current within a channel whose radius is 4 *μ*m. The electron starts its motion on axis at the moment when the laser electric field is at its maximum. Figure 2b shows the corresponding electron trajectory calculated without including the radiation friction, with the magnetic boundary marked by a dashed line. The location of the magnetic boundary remains constant in this case. The normalized magnetic field at the boundary is approximately given by ${a}_{MB}\sim {10}^{-2}{a}_{0}$. The considered electron experiences very rapid dephasing, which prevents it from gaining significant energy compared to its initial value. The corresponding electron trajectory in the momentum space is shown in Fig. 2a, where the red-yellow color-scheme indicates the electron energy.

The radiation friction qualitatively changes the electron trajectory in Fig. 2b (green-blue color-scheme) and the energy exchange with the field of the laser in Fig. 2a. The radiation friction is simulated by emitting individual photons in the direction opposite to ***p*** according to the synchrotron cross-section, as detailed in Methods. It must be pointed out that the results are similar to those obtained for the continuous radiation friction used in the previous section (see ref.^59^ for a detailed comparison between the classical and QED emission processes). In particular, in the early acceleration phase, which is modeled in the previous section, the emission is apparently quasi-continuous (see Fig. 2), whence the numerical and analytical models give equivalent results. In addition, the discrete photon emission model also captures the correct physics at later times, after the electrons are accelerated to high values of *γ* and the emission is no longer continuous. As shown in Fig. 2b, the radiation friction causes the magnetic boundary to shrink. Once the boundary shrinks sufficiently to make the travel time across the channel comparable to the period of the electric field oscillations at the electron location, the electron starts gaining energy from the laser. The key feature of the energy gain process is distinctly visible in Fig. 2a, where *p*_*x*_ increases as *p*_*y*_ performs a transverse oscillation. In the absence of the magnetic field deflections, such an oscillation of *p*_*y*_ would prevent the net energy accumulation, as in the earlier part of the trajectory in Fig. 2a.

The maximum electron energy in Fig. 2a is roughly $15000\,{m}_{e}{c}^{2}$ or 7.5 GeV. Our estimate given by Eq. (23) predicts ${\varepsilon }_{e}^{{\rm{\max }}}\sim 10$ GeV for ${a}_{MB}\sim {10}^{-2}{a}_{0}$. We thus conclude that Eq. (23) captures the upper limit on the energy gain imposed by the radiation friction relatively well. It is worth pointing out that the contraction of the magnetic boundary significantly slows down once the electrons starts its rapid energy increase accompanied by a reduced phase slip, as seen in Fig. 2b. This result is in agreement with Eq. (13) that states that the shrinking of *y*_*MB*_ is determined by the phase slip Δ*ξ*. It is therefore appropriate to treat *a*_*MB*_ in Eq. (23) as a constant when estimating the energy gain.

In order to clearly identify statistically meaningful trends, we consider an ensemble of 10^6^ electrons with ${p}_{y,0}/{m}_{e}c\in [40,160]$. The electrons are uniformly distributed over the specified range of *p*_*y*,0_. The time-resolved energy spectra of the electron ensemble shown in Fig. 2c,d further corroborate the beneficial effect of the radiation friction that causes a dramatic energy increase for a large number of the electrons. The energy gain shown by a dotted line in Fig. 2d is roughly $d{\varepsilon }_{e}/dt\approx {10}^{-2}\,{\rm{GeV}}/{\rm{fs}}$. It is less than what is predicted by Eq. (15) because the electron momentum is directed forward during the acceleration with $|\theta |\ll 1$. The cutoff energy after 1500 fs is ${\varepsilon }_{e}\approx 8$ GeV (see Fig. 2e), which is again in reasonable agreement with Eq. (23).

**Figure 3:**
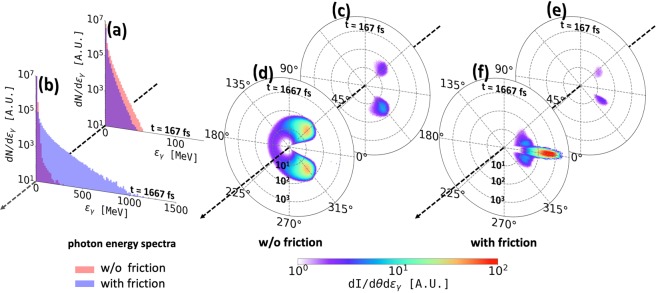
Photon emission with and without the radiation friction. (**a**,**b**) Energy spectra of the emitted photons. (**c**–**f**) Angular distribution of the emitted photons in the (*x*, *y*)-plane. The radial coordinate is the photon energy *ε*_*γ*_ on a log-scale. The polar angle is ${\theta }_{\gamma }={\tan }^{-1}({p}_{\gamma ,y}/{p}_{\gamma ,x})$.

The increase in the electron energies leads to emission of high energy gamma-rays. This aspect is illustrated in Fig. 2 by indicating the emission of photons with energies above 50 MeV (*ε*_*γ*_ > 50 MeV). These events occur only after the longitudinal momentum exceeds roughly 5000 *m*_*e*_*c*, which is the value that the electron is unable to reach in the absence of the radiation friction. The photons are emitted along ***p***, so they are highly directional because most of the electron momentum is associated with the forward motion. The time-integrated photon energy spectra for the electron ensemble reveal that while at early times the radiation friction does not cause significant differences in the emitted photon pattern (see Fig. 3a), at later times the photon spectrum with the radiation friction stretches to a cut-off energy ${\varepsilon }_{\gamma }^{{\rm{RF}}}\sim 1$ GeV, as compared to ${\varepsilon }_{\gamma }^{{\rm{noRF}}}\sim 100$ MeV if the radiation friction is neglected (see Fig. 3b). Furthermore, we find the integrated radiation signal at early times to be centered in two lobes at comparable angles ${\theta }_{\gamma }\approx \pm \,45^\circ$, irrespective of whether the radiation friction is taken into account or not (see Fig. 3c,e), whereas at late times the radiation friction leads to significant collimation of the emitted photons as compared to the case without the radiation friction in which the angular photon distribution preserves the two-lobe structure (see Fig. 3d,f).

## Summary and Discussion

We have considered the dynamics of laser-irradiated electrons in a strong quasi-static azimuthal plasma magnetic field. We have demonstrated that the radiation friction can cause a dramatic energy increase. The process consists of two subsequent stages: contraction of the magnetic boundary and enhanced energy gain from the laser field. The contraction of the magnetic boundary reduces the electron travel time across the magnetic filament thus creating the conditions necessary for the energy enhancement. The maximum energy that the electron can gain from the laser pulse before the radiation friction becomes detrimental is given by Eq. (23). In contrast to the pure vacuum acceleration, the energy increase takes place without any increase of the transverse electron oscillations. The benefit of this regime is that it leads to emission of energetic and highly-directional gamma-rays. Our results suggest that this effect could be observed at next-generation laser facilities with a laser beam of a finite width.

Although we approximate the laser pulse by an infinite plane wave, the results are robust and applicable for a pulse of reasonable duration. The electrons considered in the manuscript quickly gain a relativistic longitudinal velocity, so they are able to move with the laser pulse. We find that the electrons slide only between 30 to 50 laser wavelengths with respect to their original location within the laser pulse during the entire simulation that lasts 1667 fs or 500 laser periods. This means that our results should hold for a laser pulse with duration of 170 fs (this is a laser pulse with a wavelength of 1 *μ*m that has 50 wavelengths).

**Figure 4:**
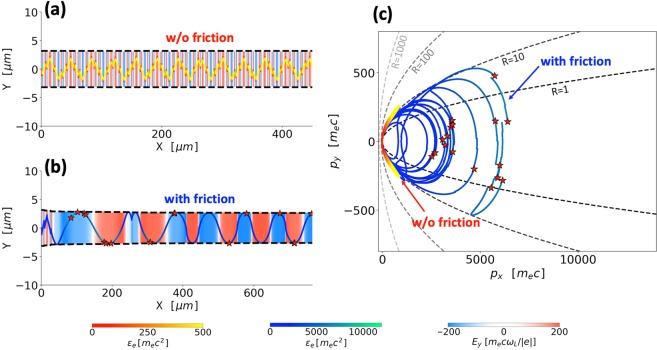
Energy enhancement by the radiation friction for an electron with *p*_*y*,0_ = 40 *m*_*e*_*c* irradiated by a laser pulse with *a*_0_ = 200 inside a magnetic filament sustained by normalized current density *α* = 4.0 defined by Eq. (5).

Our result should be viewed as an upper-limit prediction for the electron energy gain. A fully self-consistent particle-in-cell (PIC) simulation is required to determine the role of such aspects as pulse depletion, superluminal phase velocity, and transverse ponderomotive force caused by a finite beam width^60^ for specific target and laser beam parameters. One such simulation showing the energy increase due to the radiation friction is presented in ref.^61^. PIC simulations are also required to make predictions for specific laser and target parameters. The example shown in Fig. 2 is designed to qualitatively illustrate a new effect rather than to constrain laser parameters. It is important to stress that the location of the magnetic boundary and the longitudinal distance needed to achieve the energy enhancement depend on input parameters *p*_0_ and *j*_0_. In order to illustrate this point, we have performed an additional simulation for an electron with *p*_*y*,0_ = 40 *m*_*e*_*c* inside a magnetic filament with a normalized current density *α* = 4.0. The results are shown in Fig. 4. In contrast to the example shown in Fig. 2, the magnetic boundary in this case is almost two times closer to the axis, whereas the electron has to travel less than 100 *μ*m to experience a significant energy enhancement and start emitting high-energy gamma-rays.

The superluminosity is caused by the presence of the plasma, so it imposes a constraint on the electron density. The increase of the phase velocity caused by the plasma that takes into account the relativistically induced transparency can be estimated as $({v}_{ph}-c)/c\approx {n}_{e}/2{a}_{0}{n}_{c}$, where *n*_*e*_ is the electron density. This increase must be less than $(c-{v}_{x})/c$ in order to justify setting ${v}_{ph}\approx c$. We find that for our electrons we have $(c-{v}_{x})/c\approx 1.2\times {10}^{-3}$, so the corresponding condition reads $({v}_{ph}-c)/c < 1.2\times {10}^{-3}$. We have repeated the calculations presented in Sec. IV and found that the changes to the time-resolved electron spectra in Fig. 2d are indeed minimal at $({v}_{ph}-c)/c={10}^{-3}$. We thus conclude that the superluminosity plays only a secondary role in determining the energy gain if *n*_*e*_ < *n*_*c*_ for the considered laser amplitude of *a*_0_ = 200.

Finally, it is important to point out that the discussed regime of the energy enhancement has no hard threshold for *a*_0_. In contrast to the regimes that require for the Lorentz force to become comparable to the force of the radiation friction^19,62–64^, our primary requirement is that the contraction of the magnetic boundary should be sufficient during the electron interaction with the irradiating laser pulse to enable the acceleration by the laser electric field. The contraction is described by Eq. (13), so it depends not only on the amplitude of the laser pulse but also on the initial transverse electron momentum. The contraction time reduces with the increase of *a*_0_, which implies that the discussed regime can be accessed with very short laser pulses of ultra-high intensity.

## Methods

We use a fourth-order Runge-Kutta algorithm to push the particle motion under the Lorentz force with the time step $\Delta t=5\times {10}^{-4}({\lambda }_{L}/c)$ to satisfy the stringent temporal criteria not only on electron acceleration but also on photon generation. We model an infinite plane wave laser field by ${E}_{{\rm{wave}},y}={a}_{0}\,\cos \,(\xi )$ and ${B}_{{\rm{wave}},z}={a}_{0}\,\cos \,(\xi )$. The self-generated plasma magnetic field is given by ${B}_{{\rm{channel}},z}=-\,2\alpha y$. Since gamma-ray emission to occur over a short distance, the emission probability is calculated under the local constant field approximation^65–72^ by the differential emission rate of an electron with energy *ε*_*e*_ and quantum parameter $\chi ={E}_{rf}/{E}_{cr}$, where *E*_*rf*_ is the electric field in the electron’s instantaneous frame and ${E}_{cr}={m}_{e}^{2}{c}^{3}/(e\hslash )\approx 1.3\times {10}^{18}$ V/m^73^

24 $$\frac{{d}^{2}N}{d{\chi }_{\gamma }dt}=\sqrt{3}\frac{{m}_{e}{c}^{2}}{h}{\alpha }_{f}\frac{\chi {m}_{e}{c}^{2}}{{\varepsilon }_{e}}\frac{F(\chi ,{\chi }_{\gamma })}{{\chi }_{\gamma }},$$

where we used the emitted photon’s quantum parameter ${\chi }_{\gamma }=e\hslash |{F}_{\mu \nu }{k}^{\nu }|/{m}_{e}^{3}{c}^{3}$ and the fine structure constant ${\alpha }_{f}={e}^{2}/4\pi {\varepsilon }_{0}\hslash c\approx 1/137$. The radiated energy is given by^73^

25 $$F(\chi ,{\chi }_{\gamma })=\frac{4{\chi }_{\gamma }^{2}}{{\chi }^{2}}s{K}_{2/3}(s)+(1-\frac{2{\chi }_{\gamma }}{\chi })s{\int }_{s}^{\infty }{K}_{5/3}(t)dt,$$

where $s=4{\chi }_{\gamma }/[3\chi (\chi -2{\chi }_{\gamma })]$ and *K*_*n*_(*s*) are modified second order Bessel functions. Then each electron is initially assigned a final optical depth ${\tau }_{f}=\,\log \,[1/(1-P)]$, with a random number $P\in [0,1]$ modeling stochastic emission and straggling. The differential rate

26 $$\frac{d{\tau }_{\gamma }}{dt}={\int }_{0}^{\chi /2}\frac{{d}^{2}N}{dtd{\chi }_{\gamma }}d{\chi }_{\gamma }$$

is then advanced over each time step until the assigned optical depth is reached ${\tau }_{\gamma }\ge {\tau }_{f}$. In the corresponding time step, the electron emits a photon with its specific value ${\chi }_{\gamma }^{f}$ found from the relation

27 $$\eta =\frac{{\int }_{0}^{{\chi }_{\gamma }^{f}}\,F(\chi ,{\chi }_{\gamma })/{\chi }_{\gamma }d{\chi }_{\gamma }}{{\int }_{0}^{\chi /2}\,F(\chi ,{\chi }_{\gamma })/{\chi }_{\gamma }d{\chi }_{\gamma }},$$

where $\eta \in [0,1]$ is a uniformly distributed random number. Then the photon energy, $\hslash {\omega }_{\gamma }$, is determined by $\hslash {\omega }_{\gamma }=2{m}_{e}{c}^{2}{\chi }_{\gamma }^{f}\gamma /\chi$ and the electron’s momentum after the emission is given by ${\overrightarrow{p}}^{f}=[1-\hslash {\omega }_{\gamma }/(cp)]\overrightarrow{p}$. Finally, since the gamma-rays are primarily emitted within a cone of opening angle $\Delta \theta \lesssim {m}_{e}{c}^{2}/{\varepsilon }_{e}$ and we consider ${\varepsilon }_{e}\gg {m}_{e}{c}^{2}$, we assume them to be emitted along the electron’s instantaneous direction of motion.

## Data Availability

All relevant numerical data supporting our findings are available from the corresponding author upon reasonable request.
